# Physicochemical and bacteriological quality of swimming pools water in Kombolcha Town, Northeastern Ethiopia

**DOI:** 10.3389/fpubh.2023.1260034

**Published:** 2024-01-08

**Authors:** Tarikuwa Natnael, Seada Hassen, Belay Desye, Lebasie Woretaw

**Affiliations:** Department of Environmental Health, College of Medicine and Health Sciences, Wollo University, Dessie, Ethiopia

**Keywords:** physicochemical quality, bacteriological quality, swimming pool, Kombolcha Town, Ethiopia

## Abstract

**Introduction:**

The use of swimming pool water for recreation, rehabilitation, and athletics is widespread around the world, especially in large Cities and Towns. However, poorly managed swimming pool water can facilitate the spread of illnesses connected to recreational water. Despite this, there is no evidence on quality of swimming pools water in Kombolcha Town. Therefore, this study was aimed to evaluate the physicochemical and bacteriological quality of swimming pools water in Kombolcha Town.

**Methods:**

A laboratory-based cross-sectional study was conducted from February to April, 2021 in Kombolcha Town. A total of 90 water samples were collected from the three outdoor swimming pools. The collected data of both physicochemical and bacteriological parameters were entered into Microsoft Excel 2010 and analyzed using SPSS version 25.0. One-way ANOVA was used to test whether there were statistically significant differences between different swimming pools. The level of significance was declared at a *p*-value of <0.05.

**Results:**

In this study, out of all the pool water samples that were examined, 37.8% had pH values between 7.2 and 7.8, 36.7% had temperatures between 21°C and 32°C, and 26.7% had turbidity values that were within the WHO standard. Furthermore, only 16.7% of the pool water samples showed residual chlorine levels of 2–3 mg/L. In addition, only 27.8, 35.6, and 32.2% of the samples, respectively, met the WHO criterion for total coliform, fecal coliform, and heterotrophic plate count.

**Conclusion:**

The result indicates that most pool water samples did not fulfill both the physicochemical and bacteriological quality of the WHO standard limit for swimming pools. Thus, it is crucial to clean and regularly check the pool water, apply pool safety requirements, and raise pool user’s awareness about the danger of pool water pollution through training.

## Introduction

A swimming pool is a water-filled concrete tank or an artificial basin used for recreation, rehabilitation, and athletics (1). In addition to being used, swimming pool water provides a source for the spread of many dangerous microorganisms, including bacteria, viruses, protozoa and fungi, which has led to epidemics of illnesses linked to recreational water (2, 3). These diseases are transmitted through ingestion, inhalation, and contact with contaminated pool water and environment (4).

Swimming pools can become contaminated with pathogenic microorganisms that enter the pool directly or indirectly through contaminated air, soil, dust, rainwater, sewage, human or animal waste, and individual pool users (5). Pathogenic microorganisms in swimming pool water cause 3–8% risk of acute gastrointestinal illness (AGI) (6). Wading, playing or swimming in the water was observed to be a significant risk factor for AGI (7). Infections of the respiratory tract, skin, eye, outer and middle ear, giardia, cryptosporidiosis, hepatitis A, adenoviruses, and noroviruses can also be caused by contaminated pool water (3, 8–11). Besides microbial contamination, swimming pools can also become contaminated with chemicals, pharmaceuticals and personal care products, causing recreational water-associated outbreak (12, 13).

A recreational water-associated outbreak has been causing morbidity and mortality, globally. Evidence shows that in the year 2011–2012, 90 outbreaks resulted in at least 1,788 cases, 95 hospitalizations, and one death in the United States due to treated and untreated recreational water (14). Moreover, during 2000–2014, the United States reported 493 outbreaks associated with treated recreational water (15). The first and largest outbreak associated with recreational water occurred in pools, where inadequate disinfection was associated with 69% of pool outbreaks (16).

Protozoan parasites in swimming pools have contributed to the development of water-borne diseases. For example, between 2011 and 2016, at least 381 outbreaks attributed to water-borne infections of protozoan parasites were documented. Outbreaks were most commonly caused by the genus Cryptosporidium (17). Pool water pollution, lack of education and training, poor pool construction, and lack of disinfection have been linked to Cryptosporidium outbreaks (18).

The most critical actions to prevent swimming water-associated illness include a pre-swim shower, the use of a toilet before swimming, a pre-swim footbath, the use of goggles, and appropriate pool water treatment (3, 5). Moreover, raising the awareness of swimming pool operators is important in reducing the health risks in pool water. The result of the study showed that after training, the proportion of unacceptable samples dropped by 23.5% (19). Despite these measures, many pools water in various parts of the world were polluted, and the results showed deviations from normal recommendations for pool water.

A Palestine study revealed that the microbial counts for the swimming pool sample were unacceptable to the WHO standard of bacteriological limits (20). Another recent study in Nigeria illustrated that both the physicochemical and bacteriological quality of the swimming pool water were above the WHO and EPA permissible limits. The total bacterial, coliform, and *Escherichia coli* counts of the pool water samples were high (21). Studies in Ethiopia also revealed that swimming pool water was highly polluted and did not meet the WHO standards for swimming pools (22, 23).

The study findings in various regions of the world demonstrated that the standard value for swimming pool water quality is not being met by the water in most swimming pools. This increases the risk of contracting diseases, mainly gastrointestinal illnesses, for swimming pool users (6, 24–27). By its very nature, this ailment spreads to the community through swimming pool users, posing a health risk to the national population. Thus, determining the swimming pool’s water quality is crucial to give evidence-based intervention in order to protect everyone’s health in the community.

Although there have been numerous studies on the subject of swimming pool water quality in various parts of the world, there are limited studies on the subject in Ethiopia. Additionally, there are no regulations for the safe usage and quality control of swimming pool water in Ethiopia. Hence, this study may close the gap by evaluating the physicochemical and bacteriological quality of swimming pools water in Kombolcha Town. The results of this study may serve to shed light on how to enhance the water quality in swimming pools and the health of swimming pool users.

## Materials and methods

### Study area

**Figure 1:**
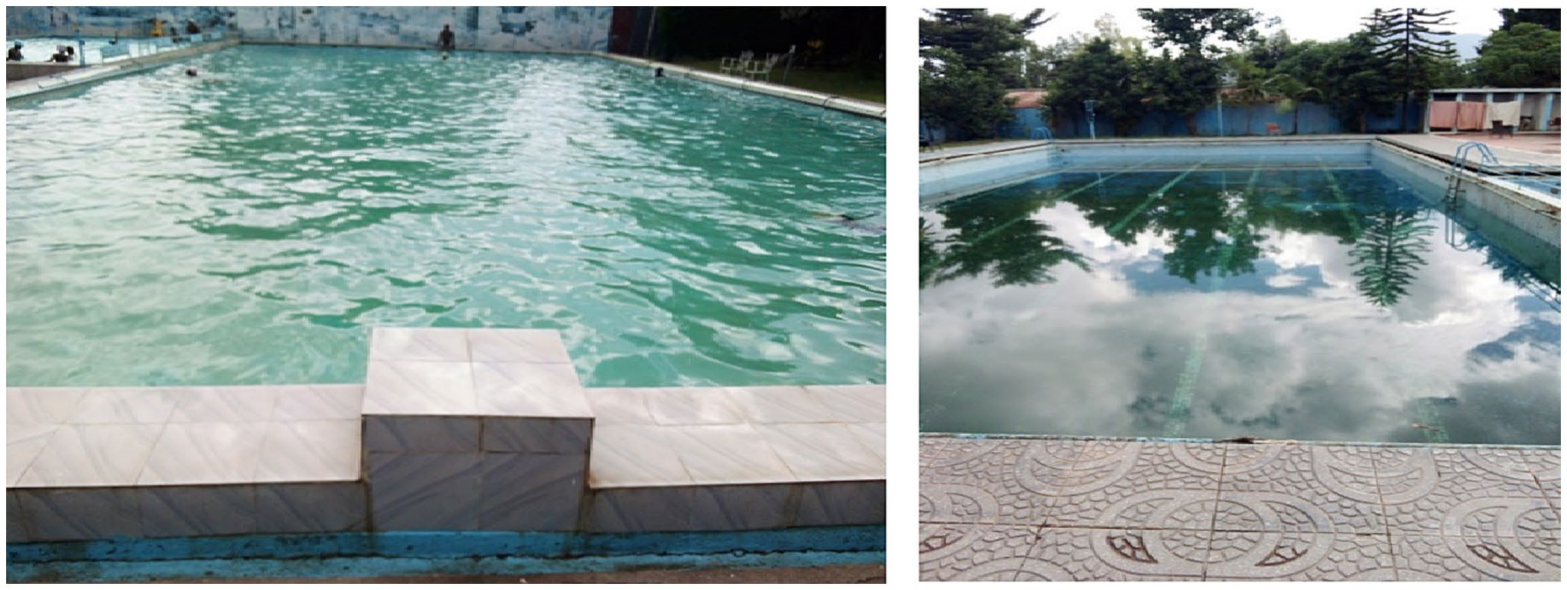
Sample photos in the study area (by primary author).

The current study was carried out at outdoor swimming pools in Kombolcha Town. The Town of Kombolcha is located in the Amhara region, 376 km from the City of Addis Ababa. It has a latitude and longitude of 11°5′N 39°44′E with an elevation between 1842 and 1915 meters above sea level. Based on the 2007 population and housing census projection, conducted by the Central Statistical Agency of Ethiopia (CSA), Kombolcha District had a total population of 116,682 in 2014. Of the total, 86,833 (74.4%) lived in urban *kebeles* and 29,849 (25.6%) in peri-urban *kebeles* (28). The Town had three outdoor swimming pools as depicted in Figure 1. These swimming pools were used for rehabilitation, recreation, and athletics. The swimming pools are used by the communities of the Town, hotel guests, and sports science students of the University of Wollo as well as visitors.

### Study design and period

A laboratory-based cross-sectional study was carried out from February to April, 2021 to determine the physicochemical and bacteriological quality of swimming pools water in Kombolcha Town.

### Sample size, sampling procedure, and sample handling

A total of 90 pool water samples (30 water samples from each swimming pool) were collected from the three outdoor swimming pools in Kombolcha Town. Swimming pool water samples for bacteriological analysis were collected aseptically using 250 mL sterile glass bottles. Additionally, a sterile 1 L polyethylene bottle was used to collect samples for physicochemical analysis. Prior to taking pool water samples for physicochemical analysis, the bottles were rinsed three times with pool water to be collected. A sample of the pool’s water was obtained by holding the sample bottle’s base at a 45-degree angle, dipping it elbow-deep into the water, then turning it right-side up to collect the sample vertically into the water at a depth of around 20 cm.

The mouth and inside of the bottles, as well as their caps, were never contacted during sample collection by fingers, clothing, or unsterile objects. Samples were taken when the maximum numbers of swimming pool users were in the pool and at the most crowded location each week (Friday, Saturday and Sunday) for a period of 2 months and 1 weeks. On removal from the pool water, collected samples were tightly closed, labeled and immediately placed in a 4°C icebox and transported to the Department of Environmental Health at Wollo University for analysis. The sampling protocol was carried out scrupulously following the standard methods of the American Public Health Association (APHA) and WHO guidelines for swimming pool water (29).

### Physicochemical analysis

Physicochemical parameters of pH, temperature, turbidity, and residual chloride were determined in all pool water samples. Sample pH and temperature parameters were measured using pretested and calibrated portable digital multi-parameter probe (HQ40d, HACH Company) at the time of sampling. The turbidity was also measured at point of collection using Wagtech International Turbidity Meter (Wag-WT3020, Halma PLC Company). Residual chloride analysis was performed in the laboratory according to the analytical methods described in Standard Methods for Water and Wastewater Analysis (29).

### Bacteriological analysis

Bacteriological quality sample analysis was performed within 6 h of sample collection using the required preservation techniques based on standard methods adopted from Standard Methods for the Analysis of Water and Wastewater (29). The most probable number (MPN) technique using multiple fermentation tubes was used to determine total coliform (TC), and fecal coliform (FC). Additionally, a plate count method was used to determine heterotrophic plate counts (HPC) in pool water.

### The most probable number technique

Presumptive coliform test was performed using single and double strength solutions of MacConkey broth (Himedia lab Pvt. Ltd, India). The first set of the tubes had 10 mL of sterile double strength broth and the second and the third sets had 10 mL of single strength broth. The three sets of tubes would receive 10 mL, 1 mL and 0.1 mL quantities of water samples using sterile syringes, respectively, and were incubated for 48 h at 37°C. Tubes showing acid and gas formation was considered presumptive coliform positive. The most probable number (MPN) was then estimated from the table for three tube test (29). Confirmatory testing was carried out by transferring a loop full of culture from each tube which showed acid and gas in the presumptive test and inoculating it in to Brilliant Green Lactose Bile (BGLB) broth (Oxoid, England). The inoculated tubes were incubated at 37°C for 48 h for total coliforms and 44.5°C for 24 h for fecal coliforms and the total coliforms and the fecal coliform count was determined from the Most Probable Number (MPN) table.

### Heterotrophic plate counts

Nutrient agar (Sisco Research Lab. Pvt. Ltd., India) was prepared according to the manufacturer’s instructions and 1 mL of the original water sample was serially diluted with 9 mL of normal saline from 10^−1^ to 10^−7^, then, using the pour plate method, 1 mL of the diluted sample was poured into sterilized petri plates in triplicate then the sterilized media was poured on the sample and incubated in inverted manner at 37°C for 48 h. After incubation, the total viable count was counted using a digital colony counter.

### Data quality control

Instrument calibration was performed prior to pool water sample analysis. Triplicate analysis of each parameter was performed for reliable results. We checked the expiration dates of various chemicals and followed the manufacturer’s instructions for media preparation. All collected swimming pool water samples were kept at 4°C before analysis. All media and materials were sterilized using an autoclave and all microbiological analysis was performed inside a biosafety cabinet. The standard methods of APHA sampling techniques and analysis procedures were used. Additionally, samples were collected by well-trained laboratory personnel and supervisors. Moreover, to assure the validity of the analysis, 1 blank per 30 samples were analyzed following the same procedure and the sterility of the prepared culture media was checked by incubating 5% representative of the batch culture at 37°C overnight and observing for bacterial growth. Generally, tight quality control measures were employed throughout the entire process of the sample collection and laboratory work, such as pre- analytical and post-analytical.

### Data management and analysis

Data collected from physicochemical and bacteriological pool water sample analysis were entered into Microsoft Excel 2010 and exported to SPSS version 25.0 for statistical analysis. Descriptive statistics including mean, standard deviations, frequencies and percentages were determined. The WHO standard for swimming pools was compared to the values of the bacterial counts and physicochemical properties of the swimming pools water samples to determine whether they were acceptable or unacceptable. One-Way Analysis of Variance (ANOVA) was used to test whether there were statistically significant differences between different swimming pools. In each case, *p* values of less than 0.05 were used to determine statistical significance.

## Results

### Characteristics of the swimming pools

Of the 3 swimming pools, pool one and two had a length of 25 m, while pool three had a length of 22 m. The swimming pools were all rectangular in design, with depths of 2.2 m, 2.8 m, and 3 m, respectively. The swimming pools ranged in volume from 687.5m^3^ to 937.5m^3^, with average daily users of 15. To treat the pool water, all swimming pools employ chlorination. They were filled with water from various sources, including tap water and ground water (Table 1).

**Table 1:** Characteristics of swimming pools in Kombolcha Town, Northeastern Ethiopia, 2021.

Swimming pools	Basin type	Volume	Average daily users	Disinfection/treatment	Source of water
Swimming pool one	Outdoor	687.5 m^3^	20	Chlorination	Tap water
Swimming pool two	Outdoor	937.5 m^3^	10	Chlorination	Ground water
Swimming pool three	Outdoor	492.8 m^3^	15	Chlorination	Tap water

### Physicochemical quality of the swimming pools water

In this study, physicochemical parameters such as pH, temperature, turbidity and residual chlorine were determined. From 90 swimming pool water samples, 37.8% (*n* = 34/90) had pH values within the WHO recommended limit of 7.2–7.8 while, 62.2% (*n* = 56/90) of the water samples had pH values out of the recommended limits. With regard to temperature, 36.7% (*n* = 33/90) of swimming pools water showed temperature values within the WHO recommended limit and 63.3% (*n* = 57/90) were out of the recommended limit (Table 2).

**Table 2:** Physicochemical compliance of swimming pools water of Kombolcha Town, Northeastern Ethiopia, 2021.

Parameters	WHO limit value	Swimming pool one (*n* = 30)	Swimming pool two (*n* = 30)	Swimming pool three (*n* = 30)	Average (*n* = 90)
pH	7.2–7.8	13 (43.3%)	7 (23.3%)	14 (46.7%)	34 (37.8%)
Temperature (°C)	21–32	12 (40%)	8 (26.7%)	13 (43.3%)	33 (36.7%)
Turbidity (NTU)	1–5	9 (30%)	4 (13.3%)	11 (36.7%)	24 (26.7%)
Residual chlorine (mg/l)	2–3	7 (23.3%)	2 (6.7%)	6 (20%)	15 (16.7%)

### Bacteriological quality of the swimming pools water

All water samples collected from the three swimming pools were examined for total coliform (TC), fecal coliform counts (FC), and heterotrophic plate counts (HPC).

### Total coliform counts and compliance

From the three pools, 90 water samples were collected for water quality analysis, 27.8% (*n* = 25/90) of the pool water samples had total coliforms below 1MPN/100 mL and 72.2% (*n* = 65/90) counted over 1MPN/100 mL (Table 3).

**Table 3:** Total coliform counts and compliance of swimming pools water of Kombolcha Town, Northeastern Ethiopia, 2021.

Swimming pools	Total number of samples	WHO acceptable limit (<1 MPN/100 mL)	WHO un-acceptable limit (>1 MPN/100 mL)
Swimming pool one	30	9 (30%)	21 (70%)
Swimming pool two	30	6 (20%)	24 (80%)
Swimming pool three	30	10 (33.3%)	20 (66.7%)
Average	90	25 (27.8%)	65 (72.2%)

### Fecal coliform counts and compliance

The fecal coliform counts were varied across the three swimming pools. In this finding, swimming pool one, swimming pool two and swimming pool three showed 56.7, 76.7, and 60% fecal coliform counts, which were unacceptable by the WHO, respectively. From a total of 90 pool water samples tested for fecal coliforms from three swimming pools, 58 (64.4%) were unacceptable by the WHO standard (>1 MPN/100 mL) while 32(35.6%) of the samples were acceptable by the WHO standard (<1 MPN/100 mL) (Table 4).

**Table 4:** Fecal coliform counts and compliance of swimming pools water of Kombolcha Town, Northeastern Ethiopia, 2021.

Swimming pools	Total number of samples	WHO acceptable limit (<1 MPN/100 mL)	WHO un-acceptable limit (>1 MPN/100 mL)
Swimming pool one	30	13 (43.3%)	17 (56.7%)
Swimming pool two	30	7 (23.3%)	23 (76.7%)
Swimming pool three	30	12 (40%)	18 (60%)
Average	90	32 (35.6%)	58 (64.4%)

### Heterotrophic plate counts and compliance

This study showed different heterotrophic plate counts in the three swimming pools. Highest HPC count was observed at swimming pool two, which was 24 (80%), while lowest count of HPC was observed at swimming pool three with 18 (60%). In this study, of 90 pool water samples tested for heterotrophic plate counts, 61 (67.8%) was above the upper limit of the WHO guideline value for HPC >200 CFU/mL and the remaining 29 (32.2%) was in line with the WHO guideline value for HPC <200 CFU/mL (Table 5).

**Table 5:** Heterotrophic plate counts and compliance of swimming pools water of Kombolcha Town, Northeastern Ethiopia, 2021.

Swimming pools	Total number of samples	WHO acceptable limit (<200 CFU/mL)	WHO un-acceptable limit (>200 CFU/mL)
Swimming pool one	30	11 (36.7%)	19 (63.3%)
Swimming pool two	30	6 (20%)	24 (80%)
Swimming pool three	30	12 (40%)	18 (60%)
Average	90	29 (32.2%)	61 (67.8%)

### Comparison of the physicochemical and bacteriological quality of the swimming pools

The mean physicochemical and bacteriological counts of pool water samples were compared across the three swimming pools using One-Way ANOVA. The ANOVA test revealed a statistically significant difference in the physicochemical parameters (pH, temperature, and turbidity) and bacteriological parameters (TC, FC, and HPC) among the three swimming pool water samples (*p* < 0.05) (Table 6).

**Table 6:** Analysis of variance table for physicochemical and bacteriological analysis of swimming pool water samples from different swimming pools in Kombolcha Town, Northeastern Ethiopia, 2021.

Parameters	Swimming pool one	Swimming pool two	Swimming pool three	*p*-value
pH	7.3 ± 0.36	8.1 ± 0.68	7.2 ± 0.28	0.000
Temperature (°C)	27.00 ± 5.42	30.90 ± 3.67	26.00 ± 5.62	0.001
Turbidity (NTU)	6.50 ± 2.09	8.00 ± 1.74	5.50 ± 2.09	0.000
RC (mg/l)	0.47 ± 0.86	0.13 ± 0.50	0.40 ± 0.81	0.191
TC (MPN/100 mL)	78.68 ± 84.61	121.33 ± 84.21	62.00 ± 79.12	0.020
FC (MPN/100 mL)	43.33 ± 70.09	73.33 ± 78.09	35.67 ± 58.77	0.030
HPC (CFU/mL)	226.00 ± 102.94	280.00 ± 94.32	214.67 ± 106.34	0.033

RC, residual chlorine; TC, total coliform; FC, fecal coliform; HPC, heterotrophic plate counts.

## Discussion

Pool water quality should be considered equally with drinking water quality, as the water has a high potential for ingestion during swimming, which can lead to water-borne illnesses (26, 27). The majority of people, however, are more concerned with the quality of drinking water and pay little attention to swimming pool water quality. Thus, the present study was conducted with the aim of evaluating the physicochemical and bacteriological quality of swimming pools water in Kombolcha Town. The current investigation found that only 29.4 and 31.8% of the samples met the WHO criterion for physicochemical and bacteriological quality, respectively.

In this study, physicochemical parameters such as pH, temperature, turbidity and residual chlorine were determined. In this finding, 37.8% of the pool water samples showed a pH value within the WHO recommended limit of 7.2–7.8. The result was similar with the study in Ethiopia, and Nigeria (23, 30) where, only 25, and 33% of the swimming pool water samples showed pH value within the WHO recommended limit. However, the finding was different from the other study in Ethiopia in which most (58.4%) of the samples had a pH value within the WHO recommended limit (31). Contrary to our finding, all samples of swimming pool water taken in Nigeria met the WHO requirement for pH (32). Moreover, there was a statistically significant difference (*p* < 0.05) between the mean pH of different swimming pools. The difference in pH value might be due to difference in the implementation of regulations, monitoring and control system. Despite this, at very low or very high pH levels, swimming pool users may have dermatitis or eye irritation due to the direct influence of the water on their eyes and skin (5). Additionally, pH plays a significant role in ensuring effective coagulation and disinfection as well as preventing damage to the fabric of the pool (1). Thus, the pH of the pool water must be monitored to ensure swimming pool users comfort, efficient disinfection and coagulation, and to avoid damage to the pool fabric.

Temperature measurement is one of the most important tests in water chemistry which control the growth of microorganisms. In this study, only (36.7%) of swimming pool water samples showed temperature values in line with the WHO recommended values (21°C–32°C). The two studies in Ethiopia showed similar result with our findings (22, 23). Contrary to the current finding, temperature measurements of all swimming pool water samples were within the WHO standard in Nigeria (33). The difference may be due to different body temperatures of pool users and weather condition. Differences in pool water temperature may also be due to different sampling seasons. There was also statistically significant difference (*p* < 0.05) between the mean temperatures of different swimming pools water. This can be due to the bathers’ load and the location of the pool which is away from sun-shading structures.

Turbidity of water is often associated with the presence of dust, microorganisms such as bacteria and other parasites in the water. In the present study, 73.4% of the pool water samples showed turbidity values above the WHO recommended values (>5NTU). The result is similar to the studies conducted in Ethiopia, where 79 and 75.9% of the pool water samples showed turbidity values above the recommended limits (22, 23). This high turbidity in our study may be due to the nearness of the main road to the swimming pools and the surrounding soil, dust, and, pollen. It can also be from colloidal and organic material downloaded by pool users while swimming. There was statistically significant difference (*p* < 0.05) among the mean turbidity values in water samples of different swimming pools water. Thus, health education programs on hygienic swimming practice must be enhanced to improve the quality of the swimming pools water.

As for residual chlorine, only (16.7%) of the pool water samples had residual chlorine level within the standard (2–3 mg/L). The result of the study in Addis Ababa and Oromia region also showed 25% compliance to the standard (22). This finding was also in line with a study conducted in Egypt, where just 20% of swimming pool water samples displayed residual chlorine levels within the acceptable range (34). Different results were observed in Bahir Dar and Palestine, where all (100%) of the pool water samples were less than the WHO standard (23, 35). This low residual chlorine may be due to the pool water not being adequately disinfected due to the high cost of chlorine. The presence of high levels of organic matter in swimming pools can also be responsible for low residual chlorine levels. Thus, proper disinfection and frequent inspection of the swimming pools’ water is highly desirable.

The microbial quality of the swimming pools water determines the health risk of the swimming pool users after swimming. In this study, 72.2% of pool water samples were above the WHO standard limit of total coliform (TC). In comparison to our investigations, 81.5% of the Ethiopian sample showed TC higher than the WHO threshold value (23). In contrast to our finding, the proportion of pool water samples that deviated from the WHO standards was significantly lower, including 20.9, 9.0, and 10.3% in Ghana, Iran and China, respectively (36–38). Their findings support the significance of on-going pool monitoring and proper upkeep. The difference may be due to variations in pool monitoring and pre-swim hygiene practices. This difference may also be due to the difference in the water sources for swimming pools. Therefore, action is urgently needed to maintain the quality of the swimming pool water through continuous monitoring, proper treatment, awareness-raising, and binding procedures.

The WHO guidelines for water quality recommend no fecal coliform in the water. However, the microbiological analysis of the pools water showed that most (64.4%) of the samples had a high occurrence of fecal coliform. Water samples from the swimming pool two are highly polluted (58.34%) compared to the other swimming pools water. Similar to our findings, significantly high counts (up to 250 MPN/100 mL) of fecal coliforms were analyzed in swimming pools water samples in Addis Ababa and Oromia region (22).

High counts of fecal coliforms were also recorded in 68% of samples in Bahir Dar (23). Additionally, in Nigeria, all pool water samples were polluted with fecal coliforms (39). The result contradicts the standard recommended values as a result adversely affects the health of the swimming pool users’. The presence of coliforms, especially FC, in swimming pools indicates fecal contamination. The high occurrence of bacteria could be related to feces released by the pool users and the result of direct animal contamination (3). In addition, lack of regular pool water quality monitoring may be a possible explanation for the presence of bacterial contamination in pool water samples. It is therefore recommended to apply pre-swim shower, toilet before swimming, regular monitoring of the pool water quality and proper chlorination of the swimming pools water.

HPC measurements in water bodies are used to gage how well the water treatment process is working and to count the amount of microbes that are re-growing. Therefore, a high level of HPC is caused by an unsuitable temperature, a lack of free chlorine disinfectant, and maybe excessive microbial growth (3). In the majority (67.8%) of swimming pools, the HPC had significant amounts of bacteria (>200 CFU/mL). Comparative result was reported in Uganda, where 68% of the swimming pool water samples had higher (>500 CFU/mL) HPC (40). The finding was also supported by other studies conducted in Bahir Dar (23) that revealed 80% of samples having HPC counts higher than the WHO recommended limit. However, the result was different from another finding in Ethiopia, where only (26.7%) of samples deviated from the WHO recommended HPC (31). This difference may be due to differences in the pool water treatment system, leaching during the rainy season, runoff, bathing load and pre-swim hygiene practices.

Moreover, in terms of TC, FC, and HPC, the One-Way ANOVA test revealed a statistically significant difference between the Kombolcha Town swimming pools. When compared to the three swimming pools, pool two had the highest readings. The reason could be due to poor pre-swim hygiene of swimming pool users and improper treatment of the swimming pool water. The study, which was done among Kombolcha town swimming pool users, also found poor pre-swim hygiene practices (41). Thus, pre-swim hygiene measures should be promoted to all pool users, such as toilets before swimming to minimize urination in the pool and accidental fecal releases. Additionally, pre-swim showering should be practiced to remove traces of urine, fecal matter and other potential water contaminants. Moreover, the surfaces of the swimming pool and the surrounding areas of the pool should be kept clean. Furthermore, direct animal access to the pool should be prevented, appropriate disinfectant concentrations should be maintained, and the pool water should be regularly monitored.

### Limitation of the study

This study did not evaluate the effects of seasonal changes in swimming pool water quality.

## Conclusion

Swimming pool water quality is essential for the health and wellbeing of the swimming pool users. The current study examined the physicochemical and bacteriological quality of swimming pools water. In this investigation, the majority of the pool water samples did not meet the WHO’s physicochemical parameters (pH, temperature, turbidity, and residual chlorine) and bacteriological quality criterion (TC, FC, and THC). Thus, good pre-swim hygiene practices are required and should be supported through appropriate hygiene education. Also, the regular treatment, checking the pool water quality and implementing pool safety guidelines are highly desirable to prevent contamination of the pool water and reduce water-borne diseases among swimming pool users. Moreover, regulations on swimming pool water quality control must be developed at the national level and must be strictly enforced. Finally, future studies should include the impact of seasonal changes in swimming pool water quality.

## Data availability statement

All data supporting the findings of this study are included in the article; however, details of the full data may be obtained from the corresponding author on reasonable request.

## Ethics statement

The Institutional Ethical Review Committee of Wollo University’s College of Medicine and Health Sciences provided the ethical approval letter with the issue number of CMHS/342/13/13. Before the study time, the Kombolcha Town Health Department gave supportive letter. The pool managers were addressed in a letter. Prior to sample collection, managers of the swimming pools were informed of the study’s intent in detail, and their consent was acquired. Information obtained from the swimming pools would be kept private; it was also made known to those in charge of the leisure areas.

## Author contributions

TN: Conceptualization, Data curation, Formal analysis, Funding acquisition, Investigation, Methodology, Project administration, Resources, Software, Supervision, Validation, Visualization, Writing – original draft, Writing – review & editing. SH: Funding acquisition, Investigation, Methodology, Project administration, Resources, Software, Supervision, Validation, Visualization, Writing – original draft, Writing – review & editing. BD: Data curation, Methodology, Project administration, Software, Supervision, Validation, Visualization, Writing – review & editing. LW: Formal analysis, Project administration, Validation, Writing – review & editing.
